# Application of Broccoli Leaf Powder in Gluten-Free Bread: An Innovative Approach to Improve Its Bioactive Potential and Technological Quality

**DOI:** 10.3390/foods10040819

**Published:** 2021-04-09

**Authors:** Urszula Krupa-Kozak, Natalia Drabińska, Natalia Bączek, Kristýna Šimková, Małgorzata Starowicz, Tomasz Jeliński

**Affiliations:** Institute of Animal Reproduction and Food Research, Polish Academy of Sciences, Tuwima 10 Str., 10-748 Olsztyn, Poland; n.drabinska@pan.olsztyn.pl (N.D.); n.baczek@pan.olsztyn.pl (N.B.); simkova.kris@gmail.com (K.Š.); m.starowicz@pan.olsztyn.pl (M.S.); t.jelinski@pan.olsztyn.pl (T.J.)

**Keywords:** *Brassica*, vegetable by-product, technological properties, texture parameters, antioxidant activity, anti-ages, gluten-free diet, coeliac disease

## Abstract

In comparison to conventional bread, gluten-free bread (GF) shows many post-baking defects and a lower nutritional and functional value. Although broccoli leaves are perceived as waste products, they are characterised by a high content of nutrients and bioactive compounds. The present study evaluated the nutritional value, technological quality, antioxidant properties, and inhibitory activity against the formation of advanced glycation end-products (AGEs) of GF enriched with broccoli leaf powder (BLP). Compared to the control, gluten-free bread with BLP (GFB) was characterised by a significantly (*p* < 0.05) higher content of nutrients (proteins and minerals), as well as improved specific volume and bake loss. However, what needs to be emphasised is that BLP significantly (*p* < 0.05) improved the antioxidant potential and anti-AGE activity of GFB. The obtained results indicate that BLP can be successfully used as a component of gluten-free baked products. In conclusion, the newly developed GFB with improved technological and functional properties is an added-value bakery product that could provide health benefits to subjects on a gluten-free diet.

## 1. Introduction

Bread is a staple food that is willingly consumed all over the world every day [1]. However, for some individuals suffering from celiac disease and other gluten-related disorders (wheat allergy and non-celiac gluten sensitivity), the consumption of conventional wheat bread and other gluten-containing products is harmful [2]. In those patients, the dietary gluten proteins or, specifically, the gliadin fraction of wheat and the prolamins from barley (hordeins) and rye (secalins) can lead to deleterious health risks and complications. Nowadays, the only available treatment for gluten-related disorders is adherence to a gluten-free diet.

Gluten-free breadmaking is a process that varies substantially from conventional breadmaking—in particular, in the ingredients used, batter rheological behaviour, and overall quality of the final product [3]. Due to the absence of the continuous three-dimensional gluten network that is responsible for the rheological properties of the dough and the development of high-quality bread, gluten-free breadmaking is challenging [4]. Therefore, the production of gluten-free bread (GF) requires complex formulations, consisting of a mixture of non-gluten basic ingredients and various additives mimicking the viscoelastic properties of gluten [5], as well as diverse technological solutions. In comparison with conventional bread, a GF shows many post-baking defects, such as unattractive appearance (irregular crust surface and pale colour), poor mouthfeel and flavour, and a shorter shelf-life. Over the last decade, considerable advances were made to improve the technological and sensory quality of GF and to prolong its shelf-life [6]. However, recently, a growing number of consumers are interesting in gluten-free products characterised by improved nutritional and health-promoting quality.

Numerous studies have shown that the fruits and vegetables-based by-products contain a substantial amount of nutrients (proteins, vitamins, and minerals), as well as functional (dietary fibre) and bioactive compounds (carotenoids, phenolic compounds, and glucosinolates) [7]. Among them, phytochemicals evince important biological activities, such as antioxidant and antimicrobial properties, thus could play a role in the prevention and treatment of noncommunicable human diseases. The beneficial effects of polyphenols and the glucosinolate derivative towards the organism, including the prevention against civilisation diseases such as cardiovascular pathologies, type 2 diabetes, some types of cancer, and neurodegenerative diseases, were widely discussed in the literature [8,9,10]. For that reason, the increasing number of research focuses on the application of by-products in gluten-free products as low-cost sources of nutrients and bioactive compounds [11,12,13]. Recently, Littardi et al. [14] evaluated the impact of the addition of ground coffee parchment to GF and indicated that this by-product was able to improve the colour of this bakery product together with a significant enhancement in the antioxidant capacity and oxidative stability.

The *Brassicaceae* family includes many vegetables commonly consumed worldwide, not only traditionally for nutrition but, more importantly, for their health-promoting properties [15]. Among them, broccoli (*Brassica oleracea* var. *italica*) has acquired considerable relevance in the last few years as a “therapeutic” food, since it contains pharmacologically active substances [16,17]. Many studies have been focused on broccoli florets, which represent only 15% of the total aerial biomass [18]. While we were interested in broccoli by-products—in particular, leaves—that are seldom utilised for food. Broccoli leaves, similar to florets, are characterised by a high content of nutrients (proteins, vitamin C, minerals, and trace elements) and bioactive compounds (glucosinolates, phenolic acids, and flavonoids) [19,20]. Although perceived as a waste product, they might be consumed as a valuable fresh product or as sources of phytonutrients, allowing to obtain added-value baked products [21,22]. Thus, the valorisation of broccoli by-products and their application as the ingredient of gluten-free bakery products of potential nutraceutical properties could be one of the alternative strategies to reduce food waste [23,24]. The present study investigated the suitability and functionality of broccoli leaf powder (BLP) as a GF component based on an analysis of the nutritional value, technological quality, antioxidant properties, and inhibitory activity against the formation of advanced glycation end-products (AGEs) of the developed gluten-free bread enriched with BLP (GFB).

## 2. Materials and Methods

### 2.1. Preparation of Broccoli Leaf Powder

A BLP was prepared as described previously [24]. Briefly, undamaged leaves of mature broccoli (*Brassica oleracea* L. var. *italica*) donated by the company GEMIX (Olsztyn, Poland) were cleaned of soil residues, washed with water, then blanched shortly (1 min) in hot water to inactivate enzymes and decrease the microbial load. Afterward, petioles and main midribs were removed, and leaf blades were freeze-dried, since it is a method that preserves the nutritional and biological value and the colour of the raw material [25]. Dry leaves were ground and sieved to obtain homogenous powder (particle size ≤ 0.60 mm). The obtained BLP was packed in a sealed plastic box and kept in a refrigerator for further analysis and application in experimental GF formulation.

### 2.2. Preparation of Experimental Gluten-Free Bread

In this study, an optimised GF formula [26] was used as a control (GFC). Corn starch (HORTIMEX, Konin, Poland), potato starch (PPZ “Trzemeszno” Sp. Z o.o., Trzemeszno, Poland), sugar, fresh yeast (Lesaffre Polska S.A., Wołczyn, Poland), pectin (E 440(i), ZPOW Pektowin, Jasło, Poland), rapeseed oil “Kujawski” (ZT “Kruszwica” S.A., Kruszwica, Poland), salt, and water were the main ingredients of GFC (Table 1). Previously characterised BLP [24] was incorporated into the GFB by replacing 5% (*w/w*) of corn starch in the GFC formula. This level of substitution was based on a preliminary study that showed that 5% was the acceptable replacement level that did not affect the sensory properties of bread, whereas the GFB with 7% BLP had too intense cabbage flavour (data not shown).

To prepare GFs, all solid ingredients were mixed for 5 min at minimum speed using a KitchenAid Professional K45SS mixer (KitchenAid Europa, Inc, Brussels, Belgium) in the stainless-steel bowl with a flat beater. Yeast, salt, and sugar were dissolved in the water and added to the dry mixture, together with oil. The batter was mixed for 12 min at speed 2. Then, a 240-g sample of the resulting batter was placed in a greased hexagon-shaped bread pan (10 cm × 10 cm × 9 cm length, width, and height, respectively) and proof for 40 min at 35 °C and 70% humidity. Experimental GFs were baked for 30 min at 220 °C in the laboratory oven (AB model DC-21, SVEBA DAHLEN, Fristad, Sweden). Nine loaves were baked from each formula. After baking, all bread loaves were cooled for at least 2 h at room temperature. Then, GFs were packed in clip-on plastic bags and kept in the dark at room temperature for further analysis. Products of two independent batches, fresh (2 h after baking) and/or stored (24 and 72 h after baking), were analysed.

### 2.3. Characteristics of Experimental Gluten-Free Breads

#### 2.3.1. Determination of Proximal Chemical Composition and Energy Value

The basic chemical composition was determined in freeze-dried GFs according to the standard method [27]: moisture content was analysed using the drying method (AOAC 925.10), proteins content was determined with the Kjeldahl method (N × 6.25 for nitrogen to protein conversion) (AOAC 979.09), and fat content using Soxhlet extraction with hexane (AOAC 923.03); total ash was determined using the gravimetric method by burning in a muffle furnace at 550 °C for 10 h (AOAC 923.03). The total carbohydrate content was calculated by subtracting the values of the moisture, protein, fat, and ash content from 100. The energy values (kJ) were calculated by multiplying the amount of macronutrients by the corresponding conversion factors (17 kJ/g for protein, 37 kJ/g for fat, and 17 kJ/g for carbohydrates) [28]. The conversion factor for calories calculation is 1 kJ = 0.239 kcal.

#### 2.3.2. Determination of Physical Parameters

The weight of GFs was evaluated using a digital balance with 0.01-g accuracy. The loaf volume was determined using a modified standard rapeseed displacement method, in which millet seeds were used instead of rapeseed. The specific volume (SV) was calculated as a loaf volume divided by its weight. Density (D) was calculated as a loaf weight divided by its volume. Bake loss was calculated as indicated in Equation (1).

(1) $$Bake\;loss\;\left(\%\right)=\frac{\left(a-b\right)\times 100}{a}$$

where:

*a*—the initial weight of batter before baking (g), and
*b*—the weight of baked and cooled GFs (g).

The crust and crumb colour of GFs was evaluated using a HunterLab ColorFlex (Hunter Associates Laboratory, Inc, Reston, VA, USA). Crust colour was determined at the middle point of the top of the loaf crust, while crumb colour was analysed at the middle point of the central 2-cm slice. The measurements were performed through a 3-cm diameter diaphragm containing an optical glass. The colour was expressed in accordance with the CIELab system, and the parameters determined were: lightness (L* = 0 (black) and L* = 100 (white) and chromatic components: a* (−a* = greenness and +a* = redness) and b* (−b* = blueness and +b* = yellowness). Values were the mean of at least nine replicates. 

**Figure 1:**
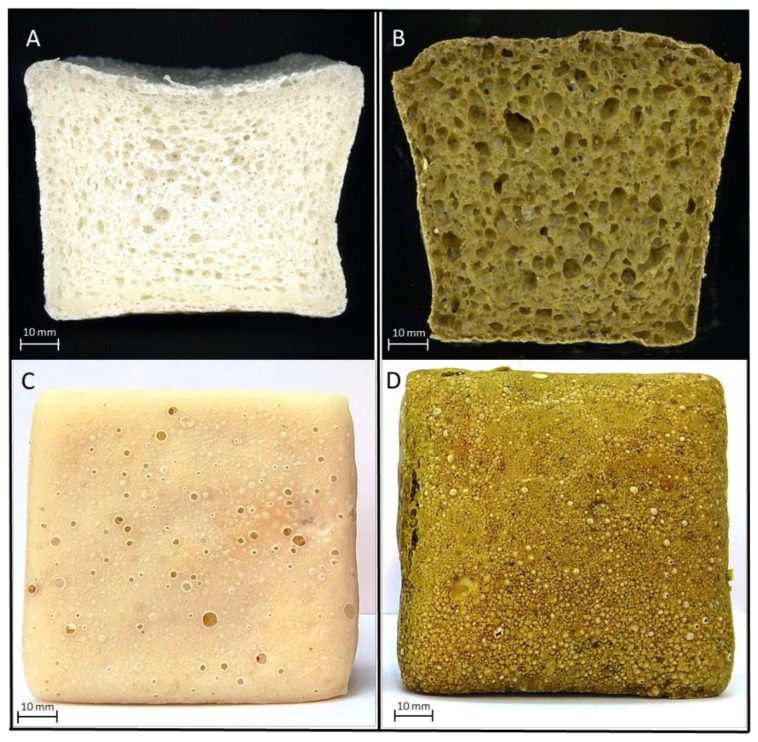
The visual appearance of crumb and crust of exemplary control gluten-free bread (**A**,**C**) and gluten-free bread with broccoli leaves powder (**B**,**D**).

To present the appearance of crumb and crust of exemplary GFC and GFB scans of the example central slice of each experimental, GF was made using a flatbed scanner (Epson Perfection V200 Photo) supported by Epson Creativity Suite Software Images (Figure 1).

#### 2.3.3. Evaluation of Textural Properties

The texture profile (TPA test) of fresh (2 h) and stored (for 24 and 72 h after baking) crumbs of GFs were analysed using a TA.HD Plus Texture Analyser (Stable Micro Systems Ltd., Godalming, UK) equipped with a 30-kg load cell. The middle bread slices of 25-mm thickness underwent a double compression cycle up to 40% deformation of its original height with a 35-mm flat-end aluminium compression disc (probe P/35). The selected settings were as follows: pre-test/test/post-test speed, 2.0 mm/s, relaxation time, 5 s, force, 10 g, and trigger, mode auto. Each slice was compressed twice to give a two-bite texture profile curve [29], from which the following textural parameters were obtained: hardness, springiness, chewiness, cohesiveness, and resilience, as calculated by the software of the texturometer. Six replicates were analysed for each kind of fresh and stored GF.

### 2.4. Evaluation of the Antioxidant Capacity of BLP and GFs

#### 2.4.1. Determination of Total Phenolic Content

The total phenolic content (TPC) was determined with the use of the Folin–Ciocalteu reagent based on the method described previously by Horszwald and Andlauer [30]. Methanol extracts were obtained from 200 mg of freeze-dried GF and 100 mg of BLP with 1 mL of 67% methanol. Samples were subjected to ultrasonic vibration (30 s) and vortexing (30 s), then were centrifuged for 10 min at 13,000 rpm at 4 °C. The above step was repeated five times, and the supernatants were collected into a 5-mL measuring flask. Methanol extracts were prepared in triplicate. The TPC assay was performed in microplates, and aliquots of 15 μL of methanol extracts were placed in microplate wells. Subsequently, 250 μL of the Folin–Ciocalteu reagent (previously diluted with water 1:15, *v*/*v*) was added, and the mixture was incubated for 10 min in dark at room temperature. Then, 25 μL of 20% sodium carbonate was added to each well, and the mixture was incubated for 20 min. The microplate was shaken automatically before reading, and absorbance was measured at λ = 755 nm with the Infinite M1000 PRO plate reader (Tecan Group AG, Männedorf, Switzerland). Gallic acid was used for standard calibration (0.03–1.0 mg L^−1^), and the results were expressed in mg of gallic acid equivalents (GAE) per one gram of dry matter (g DM) of GFs or BLP.

#### 2.4.2. Trolox Equivalent Antioxidant Capacity by ABTS Assay

The Trolox Equivalent Antioxidant Capacity (TEAC) by the 2,2′-azino-bis(3-ethylbenzothiazoline-6-sulfonic acid (ABTS) assay was performed as described by Horszwald and Andlauer [30]. To obtain an ABTS radical cation (ABTS·^+^) solution with an absorbance value of 0.70 ± 0.02 at 734 nm, 10 mL of 7-mmoL/L aqueous solution of ABTS and 0.5 mL of 51.4-mmoL/L^−1^ aqueous solution of K_2_S_2_O_4_ were mixed, then stored in the dark at room temperature for 16 h. Next, the ABTS·^+^ solution (1480 µL) was added to 20 µL of methanol extracts of BLP and GF. For the analysis in the microplates, aliquots of 10 μL of sample (the methanol extracts of BLP or GF prepared as described above for the TPC assay), standards, or blanks were placed in microplate wells. The reaction and time measurements were started upon the addition of 270 μL of the ABTS·^+^ solution. The reaction was carried out at 30 °C in dark for 6 min. After the reaction, the absorbance was measured at 734 nm with a microplate reader. Trolox was used for standard calibrations (0.25–1000 μmol/L^−1^), and the results were expressed in μmol Trolox g^−1^ DM of GFs or BLP.

#### 2.4.3. Trolox Equivalent Antioxidant Capacity by DPPH Assay

The TEAC by 2-diphenyl-picryl-hydrazyl (DPPH) radical scavenging assay was performed according to Horszwald and Andlauer [30]. To obtain the DPPH solution absorbing in the range from 0.95 to 1.10 at λ = 517 nm, 10 mg of DPPH was dissolved in 250 mL of 80% methanol. The DPPH solution was freshly prepared before analysis. For analysis, 20 μL of methanol extracts of BLP and GF (described in Section 2.4.1), blanks or standard were placed into microplate wells, and then, 300 μL of DPPH· solution was added. The reaction was performed at ambient temperature for 30 min in the dark. Trolox was used for standard calibration (0.005–0.75 mM), and results obtained were expressed as μmol Trolox Equivalents (TE) per g DM of GFs or BLP.

#### 2.4.4. Photochemiluminescence Assay

A photochemiluminescence (PCL) assay was performed as described by Zieliński, Zielińska, and Kostyra [31]. This method was used to measure the antioxidant capacity of BLP and freeze-dried GF extracts against superoxide anion radicals generated from the luminol photosensitiser under exposure to UV light in the Photochem apparatus (Analytik Jena, Leipzig, Germany). Antioxidant activity was analysed with ACW (hydrophilic condition) and ACL (lipophilic condition) kits according to the manufacturer’s protocols. For ACW, a 50-mg sample was extracted with 1 mL of water, and for ACL—a 50-mg sample was extracted with 1 mL of the MeOH and hexane mixture (4:1; *v*/*v*). The concentration of the extract solution was adjusted to ensure that the generated luminescence was within the range of the standard curve. Antioxidant capacity was calculated by comparing the delay time of the sample with the Trolox standard curve, and it was expressed in μmol Trolox g^−1^ DM.

### 2.5. Evaluation of Inhibiting Activity Against AGEs

The inhibiting activity against advanced glycation end-products (AGEs) was assessed using two in vitro model systems: bovine serum albumine (BSA)-glucose and BSA-methylglyoxal (MGO). The extraction and incubation procedures were adopted from Szawara-Nowak et al. [32]. Briefly, 150 mg of freeze-dried sample was extracted with 67% methanol by shaking at 25 °C for 40 min using a thermomixer (Thermomixer, Eppendorf, Poland). The supernatant obtained after the centrifugation was evaporated to dryness under nitrogen, and the dry residue was dissolved in phosphate buffer (0.1 M, pH 7.4). 0.5 mL of the obtained solution was incubated with 1 mL of the mixture containing BSA (10 mg/mL) and sodium azide (0.1 mg/mL) in phosphate buffer (0.1 M, pH 7.4) and appropriately D-glucose or MGO. For the measurement, 250 µl of the reaction mixture was placed into wells (microplate 96-wells, black, Porvair). The fluorescent intensity of λ_excitation_ 330 nm and λ_emission_ 410 nm (BSA-glucose), and λ_excitation_ 340 nm and λ_emission_ 420 nm (BSA-MGO) were measured. For each extract, the test was run in triplicate. A 1 mM of aminoguanidine was used as a positive control. The results were presented as a percentage of AGEs inhibitory activity.

### 2.6. Statistical Analysis

Unless otherwise stated, the data reported in all the tables are mean values and standard deviations of triplicate observations. Generally, the differences between experimental GFs were analysed with an unpaired *t*-test with Weich’s correction (*p* < 0.05), except for the differences between GFs caused by storage time that was analysed with the one-way ANOVA, using GraphPad Prism version 8.0.0 for Windows, GraphPad Software (San Diego, CA, USA).

## 3. Results and Discussion

### 3.1. Proximal Chemical Composition and Energy Value of Experimental Gluten-Free Breads

The BLP applied in the present study was previously characterised in terms of the proximal chemical composition and the profile of bioactive compounds [24] and was shown to be a good source of proteins. Additionally, a recent study by Sedlar et al. [7] indicated that, among the analysed vegetable byproducts, the broccoli leaves were characterised with the highest content of protein. In comparison with a GFC, the incorporation of BLP into the GFB resulted in a significant (*p* < 0.05) increase in the protein content (Table 2); however, in practical terms, it was a relatively small increase (1.16 g/100 g). Besides proteins, BLP was abundant in mineral compounds [24]; therefore, a significant (*p* < 0.05) enrichment in minerals was determined in experimental GFB, compared with GFC (Table 2). The obtained results are in agreement with the study by Ranawana et al. [33], who investigated the effect of the addition of the freeze-dried vegetable powder on the nutritional and physicochemical properties of wheat bread. The authors indicated that the addition of freeze-dried broccoli significantly (*p* < 0.05) increased the protein, fat, and total mineral contents in oil-free wheat bread. According to Betoret and Rosell [34], the particle size of vegetable powder affects significantly its physicochemical properties. The concentration of macronutrients (proteins and fat) in the powder of *Brassica napobrassica* leaves progressively increases as the particle size was reduced (<125 μm); conversely, a fraction of larger particle size (>1 mm) was abundant in dietary fibre. Broccoli leaves used in the present study, after being freeze-dried and ground, were sieved to obtain a homogenous powder of average particle size below 0.60 mm. Therefore, even if the physical properties of BLP could have an impact on the nutritional value of the enriched product, BLP could be recommended as an ingredient enriching GF in nutritional compounds, as similarly indicated by Sedlar et al. [7]. The energy value of GFB was higher than that of unsupplemented GFC (Table 2), mainly due to a higher fat content delivered by BLP [7]. Broccoli leaves are a rich source of polyunsaturated fatty acids, mainly α-linolenic, linoleic, and palmitic acids [35,36], which is their additional important nutritional benefit. However, the profile of fatty acids was not analysed in this study and requires further confirmation.

### 3.2. Technological Parameters of Experimental Gluten-Free Bread

The effect of BLP on the technological parameters of experimental GFs is shown in Table 3. Moreover, the differences in the appearance between the GFC and GFB can be perceived in Figure 1. The specific volume of the GFC determined in the present study was similar to the results reported previously [26]; however, in comparison with wheat bread, the value of this parameter was meaningfully lower [37]. A specific volume of a conventional wheat bread ranged from 3.5 to 5.5 cm^3^/g [38,39], while its value for GF was meaningfully reduced and fluctuated around 2 cm^3^/g, depending on the ingredients used [26,40]. The use of BLP in the experimental GF formulation influenced the technological parameters of GFB. Compared with a GFC, the specific volume of GFB rose by approximately 30% (Table 3). Besides that, a significant decrease in the bake loss was detected in GFB. The specific volume is one of the most important technological parameters of bread quality; however, it cannot be considered as the most important quality factor itself. In breads baked in pans, high values of specific volume, usually associated with proper aeration of the bread loaves, are required to obtain products able to satisfy the consumers [41]. Therefore, the appropriate gas bubble entrapment together with stabilisation of the foam structure are also essential to achieve an acceptable texture, in which the resulting pores should be small, regular, and spread regularly across the crumb. On the other side, changes determined in both parameters could result from the BLP characteristics as physical parameters of bread depending on the type and amount of protein used in dough formulation, as well as on its interaction with starch. A recent study by Sedlar et al. [7] demonstrated that proteins obtained from broccoli leaves exhibited important functional properties, including a high solubility in the alkaline condition, favourable emulsifying abilities, and water absorption capacities, as well as foaming capacity and stability. Therefore, it is possible that BLP, due to high protein content, could influence the stability of the batter during baking. Consequently, it is possible that proteins of BLP could potentially form a stable network, somewhat mimicking gluten properties. However, the study by Ranawana et al. [33] showed contrary results, indicating that wheat bread with freeze-dried broccoli powder (10%) exhibited a poor degree of leavening and was, therefore, the smallest, compared with loaves of bread with other vegetable powders. The authors explained the reduced volume of broccoli bread by the activity of enzymes present in the cruciferous vegetables [42]. Whilst in the present study, the BLP was prepared from thermally pretreated leaves (blanched). Thus, these enzymes were inactivated, creating optimal conditions for yeast fermentation that resulted in the improvement of the technological quality of GFB.

The results of the instrumental colour analysis of experimental GFs are presented in Table 3. The application of BLP influenced significantly (*p* < 0.05) all the analysed parameters of colour in the experimental bread. Both the crust and crumb of GFB were much darker (50.41 and 34.92, respectively) than the crust and crumb of GFC (75.89 and 71.58, respectively), which were pale and whitish. The crust and crumb colour strongly influence consumer choices [43]. Therefore, the darkening of starchy GFs is desirable and beneficial, as usually, they tended to have a light-coloured crust [26] that, in comparison with wheat flour counterparts, is perceived as unattractive. The visual colour difference between the typically creamy GFC and greenish-brown GFB (Figure 1) was evidenced by a colorimetric analysis. Contrary to the positive a* value indicating a slightly reddish colour of the GFC, a negative value of this coordinate was determined for the crust (−3.65 ± 0.31) and crumb (−1.47 ± 0.14) of GFB, indicating its greenness. The values of the b* coordinate were positive for both experimental GFs; however, GFB—in particular, its crust—was significantly more yellow than GFC (Table 3). The differences in the colours determined between the experimental GFs resulted from applied freeze-dried BLP, which was characterised with an intensive green hue (a* = −9.10 ± 0.03; b* = 27.67 ± 0.14). Among different techniques of dehydration, freeze-drying contributes to the preservation of colour and appearance and to minimise the degradation of thermolabile compounds, many of them responsible for the aromas and nutritional value of vegetables [44]. Many studies have demonstrated that the use of pigmented by-products of vegetable processing in bakery gluten-free products affected the colour parameters of the final product [12,13]. Therefore, it was expected that the green BLP applied in the present study would confer the colour characteristics of the supplemented GFB. Similarly, our previous study, where BLP was used to partly replace corn and potato starches in gluten-free sponge cake, resulted in a vivid-green end product [23]. However, in the confectionery product, the vividly green colour of the BLP-supplemented sponge cake was maintained mainly due to the high presence of sugar, while in GFB, since the content of sugar was much lower, a more brownish product was obtained.

### 3.3. Textural Properties of Fresh and Stored Experimental Gluten-Free Bread

The texture profile of crumb of fresh (two hours after baking) and stored (24 and 72 h) experimental GFs is presented in Table 4. Fresh GFC and GFB were similarly soft (13.21 and 13.80 N, respectively); however, fresh GFB was significantly (*p* < 0.05) springier and more cohesive than GFC. Besides, the chewiness of the GFB was over 50% higher compared with the GFC (Table 4). The chewiness informs about the time required to mastication a piece of food before it is swallowed. The incorporation of BLP into the gluten-free formulation prolonged the chewing time for the GFB crumb.

In general, the storage influenced negatively the texture properties of GFs, independently of the BLP (Table 4). After 24 h, the crumb of experimental GFs was more than two-time harder in comparison with the fresh crumbs. Longer storage (72 h) resulted in a further significant (*p* < 0.05) increase in the hardness of the GFC and GFB. Moreover, both stored GFs were significantly less cohesive, and their resilience was lower than in the case of the fresh samples (Table 4). The application of BLP in the gluten-free formulation caused a significant reduction of crumb springiness; thus, the GFB became very crumbly. However, in comparison with fresh GFB, the chewiness of stored crumb did not change meaningfully, contrary to the GFC stored for 72 h (Table 4). Ranawana et al. investigated the effect of the addition of freeze-dried vegetables (carrot, tomato, beetroot, and broccoli) on the storage properties of wheat bread with [45] and without oil [33]. The authors indicated that, among analysed vegetable breads, the broccoli bread was significantly (*p* < 0.05) harder compared to the control wheat bread both on the day of baking and during storage. However, the deterioration in texture attributes was more pronounced in the oil-free wheat bread [33]. Typically, a GF is characterised by a compact crumb with low cohesiveness and elasticity and, thus, high brittleness [46]. The textural characteristics of GF are strongly influenced by the ingredients used. Thus, if gluten is absent, the improvers (hydrocolloids, gums, and enzymes) become an obligatory element mimicking its functions [47,48], yielding a GF of satisfactory technological quality. Among them, fat-mimetic ingredients could be considered for improving texture, sensory characteristics, and shelf-life of baked products [49].

### 3.4. Antioxidant Capacity of Experimental Gluten-Free Bread

The results of the antioxidant capacity of the BLP and experimental GFs are presented in Table 5. The GFC was characterised by a relatively low antioxidant activity evaluated using all assays. Contrary, the BLP was found as a good source of TFC, consequently exerting a high antioxidant capacity. Freeze-drying, which was used to prepare BLP, is a well-known method that allows preserving the nutritional value of the starting material, including bioactive compounds [25]. Therefore, as expected, the fortification of GF with BLP significantly (*p* < 0.05) increased the antioxidant potential of experimental GFB. Among broccoli parts, leaf tissue had the highest TFC and antioxidant activity (DPPH), compared with florets and stems [19]. ABTS, DPPH, and PCL-ACW assays are associated with the activity of hydrophilic compounds like polyphenols, which have confirmation in TFC. On the other hand, the PCL-ACL assay informs about the activity of lipophilic compounds, like fat-soluble vitamins and carotenoids. The results obtained by Ranawana et al. [33,45] indicated that freeze-dried broccoli significantly increased the vitamin E (α- and γ-tocopherols) content of broccoli breads compared with the wheat bread. Moreover, the authors showed that broccoli bread contained the β-carotene and lutein that are characterised by a strong antioxidant activity. BLP was characterised by very high PCL-ACL activity, and consequently, this assay was the highest among all analysed in GF, suggesting that BLP can be a good source of lipophilic compounds, as similarly suggested by other authors [50]. However, it was not analysed in this study and requires further investigation.

A similar finding of increased antioxidant capacity after BLP incorporation was obtained in our previous study with BLP-fortified mini sponge cakes [24]. Moreover, the high antioxidant capacity of broccoli and its by-products was repeatedly reported in the literature [21,51]. Lefarga et al. indicated that wheat-based bread fortified with broccoli by-products was characterised by significantly increased TFC and antioxidant capacity in comparison to control bread without scarifying the sensory quality [21]. Interestingly, the authors reported that the TFC and antioxidant capacity increased after in vitro digestion, suggesting that the health-promoting potential of products fortified with broccoli by-products is even higher. Since the nutritional quality of GFs is relatively low, several successful attempts were performed aiming to improve the nutraceutical potential of these products, also including the vegetable by-products [12,52]. Our study also confirmed that underestimated by-products of broccoli processing can be a valuable additive to GF improving its nutritional and functional quality.

### 3.5. Anti-AGEs Activity of Experimental Gluten-Free Bread

**Figure 2:**
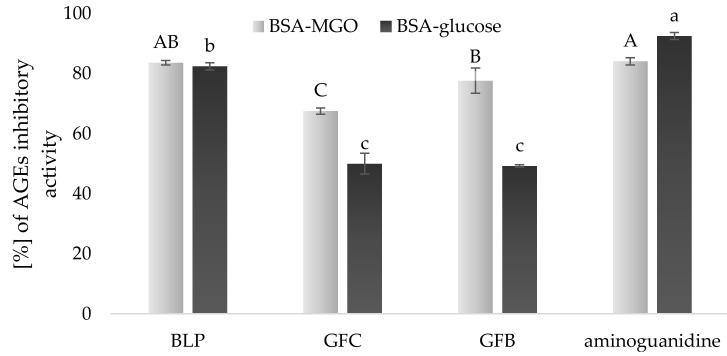
Results of anti- advanced glycation end-products (AGE) activity in models of bovine serum albumin-methylglyoxal (BSA-MGO) and BSA-glucose in samples of broccoli leaf powder (BLP), control gluten-free bread (GFC), and gluten-free bread enriched with broccoli leaf powder (GFB). The results are presented as the mean ± SD (*N* = 3). Bars with different letters denote significant differences (*p* < 0.05) when subjected to Tukey’s test.

The presence of phenolic compounds, besides the improvement of antioxidant potential, can contribute also to other bioactive activities. The advanced glycation end-products (AGEs) are formed continuously in the human body, the intensity of AGEs formation is increased by hyperglycemia and oxidative stress status [53]. Moreover, research has shown that dietary AGEs are important contributors to the pool of AGEs formed in the human body [54]. Hence, the challenge is to evaluate food products with natural inhibitors of the AGEs formation. The AGEs inhibitory activity was monitored in two model systems of BSA-MGO and BSA-glucose and presented in Figure 2. We found that extracts of BLP had high activity against the AGE formations (83.53%) in the BSA-MGO study, almost the same as the reference material of aminoguanidine (84.03%). The obtained data were in agreement with Sotokawauchi et al. [55], who noted the positive effect of broccoli sprouts decreased in the AGE formation. Additionally, a high effectiveness against AGE formation was noted in GFs after the addition of BLP (77.60%) in comparison to the control (67.47%). Therefore, the incorporation of BLP resulted in 1.15 times higher anti-AGE activity of the designed gluten-free product. In this study, we also observed that BLP showed a strong antiglycative effect (*p* < 0.05) in a BSA-glucose system, as is demonstrated in Figure 2. Similarly, in this model, the anti-AGE activity of BLP was high and accounted for 82.37%. No significant difference was observed between samples of GFC and GFB, reaching 49.97 and 49.20%, respectively.

The results obtained in this study are in agreement with other studies utilising byproducts in bread formulation to improve the anti-AGE activity. The study of Peng et al. [56] showed that the incorporation of grape seeds can reduce the level of Nε-(carboxymethyl)lysine (CML), a common advanced glycation end-product in bread. Another solution to reduce the AGEs in bread can be the application of gluten-free flour with a higher content of bioactive compounds. The study of Szawara-Nowak et al. [32] showed that buckwheat bread has higher inhibitory effects against the formation of AGEs than the control one.

Furthermore, a strong correlation was demonstrated between BSA-MGO and ACW and ACL (r = 0.988 and 0.829), whereas lower correlation coefficients were calculated for BSA-MGO vs. ABTS (r = 0.808), BSA-MGO vs. DPPH (r = 0.793), and BSA-MGO vs. TPC (r = 0.806) (Table 6). A higher positive correlation was obtained between BSA-glucose and the antioxidant activity measured by ACL, ABTS, DPPH, and r = 0.995, 0.998, and 0.999, respectively, BSA-glucose, and TPC, r = 0.998 (*p* < 0.05). A similar finding was reported by Szawara-Nowak et al. [32], who found a strong association between anti-AGE activity and TFC. Therefore, it seems that the BSA-glucose model could be a more suitable system to detect AGEs inhibitory activity of bakery products with broccoli leaves, because of high correlation coefficients between BSA-glucose and TPC values and the antioxidant activity measured by DPPH, ABTS, and ACW.

## 4. Conclusions

The present study investigated the suitability and functionality of BLP as a GF component based on an analysis of the nutritional, technological, and functional properties of the developed product. Based on the results obtained, it can be noticed that BLP can be successfully used as an additive in gluten-free bakery products. It improved the nutritional value and the technological properties of the obtained bread. In particular, the specific volume and the bake loss of GFB have been significantly improved, compared to GFC. Additionally, the crumb of fresh GFB was as soft as of the GFC, although the inclusion of BLP resulted in the deterioration of the other textural parameters. However, what needs to be emphasised is that BLP improved the antioxidant potential and inhibitory activity against the AGE formations of GFB. In conclusion, the obtained added-value baked product could provide health-promoting benefits for subjects on a gluten-free diet; however, to validate this concept and verify the positive health effects of GFB, human intervention studies are needed.

## Figures and Tables

**Table 1:** Composition of experimental gluten-free bread.

Ingredient (%)	GFC	GFB
Corn starch	36.7	31.7
Potato starch	8.9	8.9
Pectin	2.2	2.2
Sugar	2.8	2.8
Salt	0.8	0.8
Oil	1.4	1.4
Fresh yeast	2.8	2.8
BLP	-	5
Water	44.4	44.4

GFC—Control gluten-free bread, GFB—Gluten-free bread enriched with broccoli leaf powder, and BLP—Broccoli leaf powder.

**Table 2:** Macronutrients content and energy value of experimental gluten-free bread.

	GFC	GFB	*p*-Value
Moisture	55.67 ^a^ ± 0.18	55.32 ^a^ ± 0.15	0.0628
Proteins	1.22 ^b^ ± 0.04	2.38 ^a^ ± 0.09	0.0004
Ash	1.81 ^b^ ± 0.03	2.16 ^a^ ± 0.04	0.0004
Fat	0.87 ^b^ ± 0.01	2.33 ^a^ ± 0.03	<0.0001
Carbohydrates *	40.56 ^a^ ± 0.15	37.81 ^b^ ± 0.08	<0.0001
Energy value (kJ)	740 ^b^ ± 4	769 ^a^ ± 2	0.0060
Energy value (kcal)	177 ^b^ ± 1	184 ^a^ ± 1	0.0010

* Calculated from the difference. GFC—Control gluten-free bread and GFB—Gluten-free bread enriched with broccoli leaf powder. Proximate macronutrients values are g per 100 g of dry matter. Within each row, and for each factor, values with the same letter do not differ significantly (*p* < 0.05) when subjected to the unpaired *t*-test with Weich’s correction.

**Table 3:** Technological parameters of experimental gluten-free bread.

	GFC	GFB	*p* Value
Specific volume (mL/g)	2.41 ^b^ ± 0.14	3.08 ^a^ ± 0.16	0.0058
Bake loss (%)	14.96 ^b^ ± 0.09	12.07 ^a^ ± 0.63	0.0141
Crust colour			
L*	75.89 ^a^ ± 1.70	50.41 ^b^ ± 1.52	<0.001
a*	1.58 ^b^ ± 0.08	−3.65 ^a^ ± 0.31	<0.001
b*	17.28 ^b^ ± 1.12	31.95 ^a^ ± 0.94	<0.001
Crumb colour			
L*	71.58 ^a^ ± 1.70	34.92 ^b^ ± 2.81	<0.001
a*	0.35 ^b^ ± 0.11	−1.47 ^a^ ± 0.14	<0.001
b*	11.15 ^b^ ± 0.73	27.93 ^a^ ± 1.85	<0.001

FC—Control gluten-free bread, GFB—Gluten-free bread enriched with broccoli leaf powder, and BLP—Broccoli leaf powder. Within each row, and for each factor, values with the same letter do not differ significantly (*p* < 0.05) when subjected to the unpaired *t*-test with Weich’s correction.

**Table 4:** Textural properties of fresh and stored experimental gluten-free bread.

	GFC	GFB	*p*-Value
Hardness (N)			
Fresh	13.21 ^aC^ ± 1.22	13.80 ^aC^ ± 0.07	0.4905
Stored 24 h	29.53 ^aB^ ± 4.67	33.16 ^aB^ ± 4.63	0.3932
Stored 72 h	45.78 ^aA^ ± 2.55	42.86 ^aA^ ± 4.67	0.2427
Springiness			
Fresh	0.93 ^bA^ ± 0.02	0.99 ^aA^ ± 0.01	0.0196
Stored 24 h	0.90 ^aA^ ± 0.08	0.92 ^aB^ ± 0.03	0.7167
Stored 72 h	0.83 ^aA^ ± 0.01	0.89 ^aC^ ± 0.03	0.0620
Cohesiveness			
Fresh	0.55 ^bA^ ± 0.07	0.77 ^aA^ ± 0.02	0.0249
Stored 24 h	0.34 ^aB^ ± 0.11	0.44 ^aB^ ± 0.03	0.2523
Stored 72 h	0.28 ^aC^ ± 0.01	0.30 ^aC^ ± 0.01	0.0705
Chewiness			
Fresh	6.73 ^bB^ ± 1.53	10.45 ^aA^ ± 0.21	0.0496
Stored 24 h	8.88 ^aB^ ± 2.09	13.51 ^aA^ ± 2.53	0.0733
Stored 72 h	10.77 ^aA^ ± 0.91	11.51 ^aA^ ± 1.10	0.4217
Resilience			
Fresh	0.31 ^aA^ ± 0.08	0.50 ^aA^ ± 0.01	0.0523
Stored 24 h	0.16 ^aB^ ± 0.07	0.24 ^aB^ ± 0.01	0.1841
Stored 72 h	0.12 ^aC^ ± 0.02	0.13 ^aC^ ± 0.01	0.4961

GFC—Control gluten-free bread, GFB—Gluten-free bread enriched with broccoli leaf powder, and BLP—Broccoli leaf powder. ^a,b^—Within each row, and for each factor, values with the same letter do not differ significantly (*p* < 0.05) when subjected to the unpaired *t*-test with Weich’s correction. ^A,B,C^—Within each column, and for each factor, values with the same letter do not differ significantly (*p* < 0.05) when subjected to a one-way ANOVA analysis.

**Table 5:** Antioxidant capacity of experimental gluten-free bread.

	BLP	GFC	GFB	*p* Value
TFC (mg GAE/g dm)	14.42 ± 0.18	0.64 ^b^ ± 0.04	1.25 ^a^ ± 0.05	0.001
ACW (µmol/g dm)	3.29 ± 0.10	0.03 ^b^ ± 0.01	1.64 ^a^ ± 0.08	0.007
ACL (µmol/g dm)	1191.25 ± 64.37	1.05 ^b^ ± 0.04	106.97 ^a^ ± 0.87	<0.001
ABTS (µmol TE/g dm)	34.33 ± 0.29	0.13 ^b^ ± 0.01	1.77 ^a^ ± 0.06	0.003
DPPH (µmol TE/g dm)	34.11 ± 0.29	0.27 ^b^ ± 0.03	0.95 ^a^ ± 0.05	0.001

GFC—Control gluten-free bread, GFB—Gluten-free bread enriched with broccoli leaf powder, BLP—Broccoli leaf powder, TFC—Total phenolic content, GAE—gallic acid equivalents, ACW—Antioxidative capacities of water-soluble compounds, ACL—Antioxidative capacities of lipid-soluble compounds, ABTS—2,2′-azino-bis(3-ethylbenzothiazoline-6-sulfonic acid) (ABTS·^+^) radical cation-based assays, DPPT—2-diphenyl-picryl-hydrazyl (DPPH) radical scavenging assay, and TE—Trolox Equivalents. Within each column, and for each factor, values with the same letter do not differ significantly (*p* < 0.05) when subjected to the unpaired *t*-test with Weich’s correction.

**Table 6:** Correlation coefficients for bovine serum albumin-methylglyoxal (BSA-MGO), BSA-glucose and antioxidant activity, and total phenolic content (TPC) relationship.

	BSA-MGO	BSA-Glucose
ACW	0.988	0.859
ACL	0.829	0.995
ABTS	0.808	0.998
DPPH	0.793	0.999
TPC	0.806	0.998

ACW—Antioxidative capacities of water-soluble compounds, ACL—Antioxidative capacities of lipid-soluble compounds, ABTS—2,2′-azino-bis(3-ethylbenzothiazoline-6-sulfonic acid) (ABTS·+) radical cation-based assays, DPPT—2-diphenyl-picryl-hydrazyl (DPPH) radical scavenging assay, and TE—Trolox Equivalents.
